# Culture-proven endogenous endophthalmitis: ocular and systemic clinical features and outcomes

**DOI:** 10.1186/s12348-026-00593-y

**Published:** 2026-05-25

**Authors:** Charles Zhang, Mariam Tadross, Sujin Kang, Nicolas A. Yannuzzi, Thomas A. Albini, Harry W. Flynn

**Affiliations:** 1 Department of Ophthalmology, Bascom Palmer Eye Institute, University of Miami Miller, School of Medicine, 900 NW 17th Street, Miami, FL 33136 USA; 2 Miller School of Medicine, University of Miami, Miami, FL USA

## Abstract

**Purpose:**

To report the clinical presentation, systemic associations, microbiologic spectrum, management strategies, and treatment outcomes of patients with culture-proven endogenous endophthalmitis.

**Methods:**

A retrospective review of medical records was conducted of patients with culture-proven endogenous endophthalmitis between 2013 and 2024. Data collected included demographics, systemic and ocular findings, culture results, imaging studies, management strategies, and treatment outcomes.

**Results:**

A total of 50 eyes from 41 patients were identified. Of these, 30/41 (73%) presented in an outpatient setting. The most common presenting symptom was blurred vision in 36/41 (88%) with only 16/41 (39%) presenting with systemic symptoms. Vitreous cultures disclosed fungal organisms in 19/41 (46%) of cases and bacterial in the remainder. Blood cultures were positive in 23/41 (56%). A systemic source of infection was identified in 23/41 (56%), most often associated with indwelling medical devices in 7/23 (30%). Intravenous drug use was present in 7/41 (17%). All patients received intravitreal antimicrobials and 25/50 (50%) underwent vitrectomy. Death associated with systemic infection occurred in only 3/41 (7%) of patients. Evisceration was performed in 2/50 (4%) eyes. Visual acuity at last follow up was ≥ 20/400 in 26/50 (52%) of eyes.

**Conclusions:**

Endogenous endophthalmitis was associated with a fungal source in 46% of cases and was not associated with systemic symptoms in 61% of cases. In the current study, indwelling medical devices remain an important source of infection. Despite prompt treatment, visual outcomes were generally poor.

## Introduction

Endophthalmitis is classified as exogenous, resulting from direct inoculation of microbes into the eye via surgery or trauma, or endogenous from hematogenous spread of infection originating elsewhere in the body. Endogenous endophthalmitis is less common than exogenous forms, accounting for an estimated 2–8% of all cases [1–3]. The diagnosis of endogenous endophthalmitis is also clinically challenging and can be mistaken for non-infectious uveitis, overlooking an occult systemic infection [4]. This condition carries the dual threat of severe vision loss and life-threatening systemic illness, making timely diagnosis critical [5, 6]. Patients may have minimal external ocular signs early in the disease course and identifying the source of infection requires a high index of suspicion and a thorough systemic evaluation [7, 8].

Due to its low incidence and absence of prospective trials, there are no standardized evidence-based treatment guidelines for treatment of endogenous endophthalmitis. The rarity of endogenous endophthalmitis and heterogeneity of causative organisms make it difficult to formulate universal protocols, highlighting the need for more data. In practice, ophthalmologists often collaborate with infectious disease specialists to conduct systemic workups to try to identify the primary infection source.

The current study aims to help fill this gap by reviewing cases of culture-proven endogenous endophthalmitis at a large tertiary eye center with the goal of better characterizing the spectrum of presentations, management approaches, and treatment outcomes to enhance the understanding of this uncommon entity and inform future care.

## Methods

The study was approved by the University of Miami Institutional Review Board. The research adheres to the tenets of the Declaration of Helsinki and complies with the Health Insurance Portability and Accountability Act.

All patients with vitreous culture-positive endogenous endophthalmitis seen at Bascom Palmer Eye Institute (BPEI) University of Miami and Jackson Memorial Hospital between January 1, 2013 and December 31, 2024 were reviewed. Cases were identified by querying the institutional database using the ICD-10 code of H44.0X for purulent endophthalmitis. Endogenous cases were defined as those occurring without a history of ocular trauma or intraocular surgery within the preceding 6 months. Because endogenous infections arise from hematogenous seeding, the inciting organism must traverse the choroidal circulation and penetrate into the intraocular compartments; therefore, endophthalmitis was defined as infection involving the aqueous or vitreous cavity. In many cases, media opacity prevented clinical grading of inflammation, and B-scan ultrasonography was applied in these cases. To ensure inclusion of only true infectious cases and to exclude noninfectious panuveitis, only patients with a vitreous aspirate culture positive for bacteria or fungus were included.

Data collected from the medical record included age, sex, laterality, previous medical history, hospitalization, presenting symptoms, best-corrected visual acuity (BCVA) at the time of diagnosis, as well as anterior and posterior segment examination findings. Snellen visual acuity was converted to logMAR for the purposes of calculations. For patients with count fingers, hand motion, light perception (LP) or no light perception (NLP) vision, logMAR values of 2.1, 2.4, 2.7 and 3 were used, respectively, similar to prior studies [9, 10].

Management strategies were reviewed and recorded for each patient. At presentation, vitreous samples were obtained using a standard bedside vitreous tap with a 25-gauge needle. In accordance with conventional practice, anterior chamber sampling was performed when a dry tap was encountered. Systemic evaluation for the source of infection such as radiologic imaging, echocardiographic imaging, and systemic cultures were reviewed. As part of the standard evaluation for suspected endogenous endophthalmitis, HIV testing was routinely performed and was obtained for all patients included in this study. Outcome measures included the duration of follow-up, infection-related mortality, need for vitrectomy or evisceration, and BCVA at last follow-up.

No formal comparative statistical hypothesis testing was performed aside from calculating proportions, means with standard error, and medians with ranges for key variables. Given the rarity of endogenous endophthalmitis and the small sample size, the cohort was underpowered for reliable inferential comparisons, and substantial heterogeneity across cases further limited the validity of subgroup analyses. Statistical analyses were conducted using RStudio version 2024.04.2 + 764.

## Results

### Baseline characteristics

In the current study, a total of 50 eyes of 41 patients were identified. Mean age was 55 ± 3 years (range: 12–87 years) with 26/41 (63%) patients being male. In the cohort, 34/41 (83%) identified as White/Caucasian and 7/41 (17%) as Black/African American; 15/41 (37%) identified as Hispanic or Latino. Bilateral eye involvement occurred in 9/41 (22%) of patients. Previous medical history of patients included diabetes mellitus in 14/41 (34%), chronic kidney disease in 6/41 (15%), cardiovascular disease in 8/41 (20%), current intravenous drug use in 7/41 (17%), leukemia in 2/41 (5%), and chemotherapy in 2/41 (5%). Patients categorized as having leukemia or receiving chemotherapy were all undergoing active treatment for active disease at the time of endophthalmitis diagnosis. In addition, implanted medical devices included dialysis catheters in 4/41 (10%), long term bladder catheterization in 4/41 (10%), long-term intravenous lines in 6/41 (15%), and orthopedic prosthetic implants in 1/41 (2%). Overall, the patients had significant health issues with 18/41 (44%) being hospitalized in the last 6 months and 7/41 (17%) being hospitalized at the time of diagnosis. Only 6/41 (15%) had no underlying risk factor for systemic infection.

### Clinical presentation

**Fig. 1:**
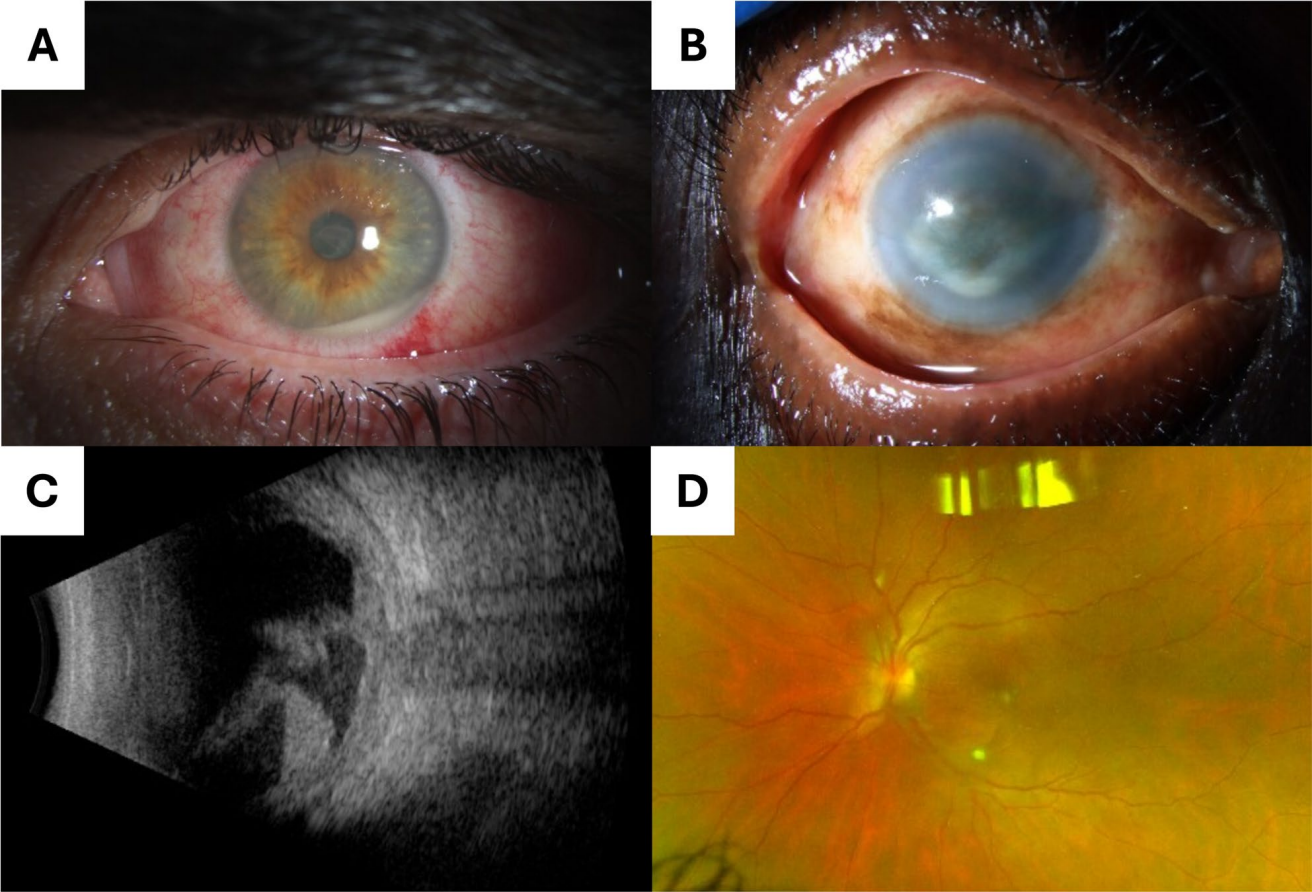
Clinical findings of patients presenting with endogenous endophthlamitis. (**A**) A 40-year-old male who presented with conjunctival injection and hypopyon from urinary catheter-associated endogenous endophthalmitis that grew *Staphylococcus aureus*. (**B**) A 79-year-old female who presented with corneal infiltrate and anterior cell fibrin that was found to have soft-tissue abscess-associated endogenous endophthalmitis that grew *Klebsiella pneumoniae*. (**C**) Ultrasonic findings of dense vitritis, thick membranes, choroidal and retinal thickening and shallow choroidal detachment of patient B. (**D**) A 31-year-old female with history of intravenous drug use presented with blurred vision. Optos ultra-widefield pseudocolor images of the fundus were performed demonstrating retinal infiltrates OS > OD. Cultures were positive for *Candida albicans*

Presenting symptoms of patients included blurred vision in 36/41 (88%), eye pain in 17/41 (42%), eye redness in 11/41 (27%), new floaters in 11/41 (27%), and photophobia in 2/41 (5%). The average duration of symptoms before diagnosis was 11 ± 2 days (range: 1–30 days). On the initial examination, conjunctival injection was present in 33/50 eyes (66%), anterior chamber (AC) cell in 31/50 (62%), hypopyon in 17/50 (34%) (Fig. 1A), AC fibrin in 11/43 (22%) (Fig. 1B), vitritis in 21/50 (42%) (Fig. 1C), subretinal infiltrates/abscess in 3/50 (6%) (Fig. 1D), and retinal detachment in 4/50 (8%). Initial visual acuity was logMAR 1.96 ± 0.13 (≈ 20/2000 Snellen equivalent; Range: 0.1-3 logMAR [20/25 to No Light Perception]). Systemic symptoms were present in 16/41 (39%) of patients, with fever present in 9/41 (22%), arthralgias in 5/41 (12%), respiratory symptoms in 3/41 (7%), digestive symptoms in 3/41 (7%), anorexia/weight loss in 4/41 (10%), headache in 1/41 (2%), malaise in 7/41 (17%), and confusion in 1/41 (2%). Mean systolic blood pressure was not elevated at 127 ± 4 mmHg (range: 95–188 mmHg), which was also true for diastolic blood pressure which was 76 ± 2 mmHg (range: 55–100 mmHg). In 5/41 (12%) cases, patients were initially given alternative diagnoses by outside providers before the diagnosis of endophthalmitis was established. These included 2/41 (5%) being diagnosed with panuveitis, 1//41 (2%) as viral retinitis, 1//41 (2%) as retinal vasculitis, and 1//41 (2%) as anterior uveitis.

### Treatment and interventions

Based on the ocular features, initial treatment included vitreous tap in all 50/50 (100%) eyes as well as intravitreal injections of vancomycin in 38/50 (76%), ceftazidime in 36/50 (72%), amikacin in 2/50 (4%), voriconazole in 27/50 (54%), and amphotericin in 2/50 (4%), and immediate vitrectomy in 3/50 (6%). Initial intravitreal antimicrobial therapy was individualized based on the patient’s clinical presentation and systemic history. Antifungal coverage was added when features suggestive of fungal infection were present, including a subacute onset, “string-of-pearls” or cotton-ball opacities within the vitreous; or systemic risk factors. The microbiologic distribution from vitreous aspirations is seen in Table 1. One eye was positive for both *Staphylococcus aureus* and *Serratia marcescens*.

**Table 1:** Organisms identified by microbiologic culture in cases of endogenous endophthalmitis. Cultures were obtained either pre-operatively during vitreous aspiration or intraoperatively from the vitreous sample in cases combined with vitrectomy

Organism	Number of Cases	% of eyes with positive cultures (50)
Candida	25	50
Staphylococcus aureus	11	22
Klebsiella pneumoniae	4	8
Coagulase negative staphylococci	4	8
Serratia marcescens	2	4
Aspergillus	1	2
Citrobacter	1	2
Escherichia coli	1	2
Listeria	1	2
Salmonella	1	2
Total	51	102*

*In one case, multiple organisms were identified, resulting in a total frequency exceeding 100%

Systemic workup to identify the underlying foci included blood cultures in all patients that were positive in 23/41 (56%) of patients. Imaging to detect the underlying foci included CT chest in 12/41 (329%), which was positive in 4/12 (33%), CT abdomen/pelvis in 13/41 (29%) that was positive in 4/13 (31%), CT head in 7/41 (17%) that was positive in 0/7 (0%), brain MRI in 10/41 (24%) that was positive in 4/10 (40%), spine MRI in 1/41 (2%) that was positive in 0/1 (0%), transthoracic echocardiography (TTE) in 25/41 (61%) that was positive in 2/25 (8%), transesophageal echocardiography (TEE) in 7/41 (17%) that was positive in 2/7 (29%). An underlying focus of infection was identified in 23/41 (56%) cases. The distribution of the underlying infectious foci is seen in Table 2. Multiple foci were identified in 7/41 (17%) patients. Among the 18 patients (44%) in whom no systemic infectious source was identified, the most common organisms were *Candida albicans* (13/18), *Staphylococcus aureus* (3/18), *Aspergillus spp.* (1/18), *Citrobacter spp. *(1/18), and *Staphylococcus epidermidis* (1/18). In these cases, due to no clear source, the most common imaging modality consisted of echocardiography which was performed in 9/18 (50%). The prominence of *Candida* among patients without an identified source likely reflects the subacute nature of endogenous fungal infection and the potential for remote seeding.

**Table 2:** Source of systemic infectious focus in patients with endogenous endophthalmitis. Systemic cultures and radiographic imaging was obtained in patients to identify the source of the infection and identified the source in 23 patients

Source	Number of Cases	% of cases with systemic focus
Medical Lines/Ports	7	30.4
Osteomyelitis	4	17.4
UTI	4	17.4
Endocarditis	3	13.0
Liver Abscess	2	8.7
Pneumonia/Empyema	2	8.7
Gynecologic	2	8.7
CNS Infection	2	8.7
Skin/Soft Tissue	1	4.3
Pyelonephritis	1	4.3
Colitis	1	4.3
Prostatitis	1	4.3
Total	30	130.4*

*In 7 cases, multiple foci were identified, resulting in a total frequency exceeding 100%

A total of 35/41 (85%) of patients were hospitalized for their systemic infection. The average length of initial hospitalization was 14 ± 1 day (range: 2–32 days). Systemic management included intravenous vancomycin in 11/41 (27%) of patients, intravenous linezolid in 1/41 (2%), systemic anti-pseudomonal cephalosporins in 6/41 (15%), systemic ceftriaxone in 5/41 (12%), systemic ampicillin-sulbactam in 2/41 (5%), systemic piperacillin-tazobactam in 1/41 (2%), systemic fluoroquinolone in 5/41 (12%), systemic triazole in 19/41 (46%), and systemic amphotericin in 2/41 (5%).

### Clinical outcomes

Mean follow-up duration was 240 ± 53 days (range: 4 days-5 years). A total of 3/41 (7%) patients died from their system infection 4, 21 and 60 days after their diagnosis, with two patients due to *Candida albicans* and another due to a *Salmonella* infection. Mean visual acuity at last follow-up was logMAR 1.31 ± 0.17 (≈ 20/400 Snellen equivalent; range, 0–3 logMAR [20/20 to NLP]) with the VA being better than 20/400 in 26/50 (52%) of eyes and 10/50 (20%) of eyes being either LP or NLP. A total of 25/50 (50%) eyes underwent vitrectomy that took place a mean of 24 ± 5 days (range: 1–90 days) after diagnosis. These vitrectomies were performed for diagnostic and therapeutic purposes. Eyes with negative bedside vitreous aspirate but known systemic infectious source underwent vitrectomy to obtain a large vitreous specimen for microbiologic confirmation. Vitrectomy was also performed to facilitate debulking of inflammatory and infectious debris. Due to documentation variability, the dominant indication could not always be assigned to a single category. No PPVs were performed for structural complications due to the absence of a rhegmatogenous component identified in cases of retinal detachment. The precise sources of vision loss could not be ascertained in most cases; however, 2/50 (4%) eyes resulted in phthisis and 2/50 (4%) underwent evisceration 21 and 60 days after diagnosis.

## Discussion

The current study confirms that endogenous endophthalmitis typically arises in patients with systemic risk factors that pose a predisposition to fungal infections. *Candida* species remained the leading cause of endogenous endophthalmitis and were isolated in 46% of patients, consistent with prior reports that have found *Candida* to be the most common causative organism [8, 11, 12]. Among bacterial cases, the most frequent isolate was *Staphylococcus aureus* in 22% of cultures which is similar to prior U.S. cohorts [2, 13]. In contrast, *Klebsiella pneumoniae*, which is the leading cause of endogenous endophthalmitis in many Asian series, often associated with hepatic abscesses, only comprised 7% of isolates in the current study [14]. The low incidence of *Klebsiella* is consistent with prior U.S. studies and reflects geographic differences in the microbiologic spectrum of endogenous endophthalmitis with gram-positive bacteria and *Candida* predominating in Western populations [15]. Comparisons with prior studies are highlighted in Table 3.

**Table 3:** Characteristics of patients with culture-positive endogenous endophthalmitis. Data collected from the current study is compared to previous similar studies

Location	Current Study (2025)	Okada et al. (1994)^2^	Schiedler et al. (2004)^3^	Wu et al. (2012)^6^
	Miami FL	Boston MA	Miami FL	Hong Kong
Most Common Organism	*Candida*	*Staphylococcus aureus*	*Candida*	*Klebsiella*
VA ≥ 20/400	52%	22%	42%	23%
(+) Blood cultures	56%	72%	26%	100%
Bilateral Disease	22%	14%	32%	5%

A majority of patients (73%) initially presented in the outpatient setting, seeking ophthalmic evaluation for ocular symptoms rather than being identified during hospitalization for systemic infection. This proportion is higher than prior series which was 43% in a prior cohort at BPEI, indicating that endogenous endophthalmitis often manifests first as an eye issue [3]. Moreover, most patients in the current study had no overt systemic symptoms at presentation. Even among those with fever, malaise or other systemic signs, these symptoms were typically mild and only elicited upon direct questioning by the examining physician. The paucity of systemic clues reinforces that endogenous endophthalmitis can occur in patients without obvious signs of sepsis, a point highlighted in prior reports [1]. Despite the subacute presentation, the time from onset of ocular symptoms to initiation of treatment in this series was 11 days, which is comparable to previous studies [1]. Only one patient experienced a protracted 30-day course prior to treatment, which occurred due to an initial misdiagnosis.

Diagnostic uncertainty remains a significant issue, particularly for fungal cases. The current study identified 12% of patients who were initially misdiagnosed. This observation is similar to prior studies that report indolent endogenous Candida endophthalmitis is frequently mistaken for non-infectious uveitis with a misdiagnosis rate as high as 50% [16]. Such diagnostic pitfalls underscore the need for a high index of suspicion, even in patients without classic systemic infection signs. It is important that endogenous endophthalmitis is considered in the differential diagnosis of patients with atypical intraocular inflammatory findings and any major risk factors for systemic disease, as delays in appropriate therapy can jeopardize both a patient’s vision and life.

Consistent with standard practice, initial management in all cases included obtaining a vitreous aspiration for culture and injecting intravitreal antimicrobial therapy targeting the presumptive organism. Systemic evaluation was pursued in parallel with blood culture in all patients. Blood cultures were positive in 56% of cases, which is notably higher than the rate of 33% reported in a previous U.S. series and more similar to a study on a South Korean population that had positive blood cultures in 81% of cases [3, 17]. There are several factors that may account for this difference. First, the present series had a smaller proportion of fungal isolates compared to prior U.S. reports. Fungal organisms are difficult to culture and may only intermittently seed the bloodstream, leading to false-negative results. In contrast, bacteremia, such as from *Klebsiella*-associated liver abscesses seen in Asian populations, can be detected by blood cultures more consistently. Second, the rate of IVDU in this cohort of 17% was lower than some prior U.S. studies, which have reported rates up to 44% [18]. Endogenous endophthalmitis in IVDU is often due to candidemia or transient bacteremias that can be episodic, potentially reducing blood culture yields.

In addition to the microbiologic work-up, all patients underwent extensive ancillary work-up to locate the primary infection source. This included TTE and TEE to evaluate for endocarditis, as well as imaging tailored to clinical suspicions, such as CT and MRI scans of specific organs. Through this approach, a source of systemic infection was identified in 56% of patients with several patients having multiple synchronous foci. Similar to previous U.S. studies, the most common source of infection in the present cohort was related to medical devices such as indwelling catheters, ports or other lines. Identification and treatment of these extraocular foci is important to reduce the occurrence of ongoing septic embolic to the eye and other organs.

In the present study, patients were treated with systemic antimicrobial therapy in addition to local intravitreal injections. The vast majority required hospitalization for intravenous antibiotics or antifungals and close monitoring. Only 3 patients died as a result of their infection, indicating better systemic outcomes than what has been previously reported. The mortality rate in earlier studies of endogenous endophthalmitis ranged from 29 to 50%, but more recent reports have demonstrated that this rate is downtrending [1, 3, 13]. Fungal endophthalmitis with candidemia, in particular, was once considered a grave prognostic indicator with one study reporting a 77% mortality rate among patients with endogenous Candida endophthalmitis in the setting of systemic candidemia [19]. In the current study, only 2 of 19 *Candida* cases were fatal. These findings suggest that outcomes have improved, likely as a result of advances in critical care and antimicrobial therapy. It is evident that developments in modern medicine have improved survival for patients with this condition, however, it still remains significantly vision-threatening with only 52% of this cohort achieving 20/400 or better vision, a number that is in line with prior studies [13].

The current study has several limitations. First, as a retrospective study, it is subject to the biases and inaccuracies inherent in medical record review. The management decisions were not standardized and were at the discretion of the treating physicians. Second, our data are drawn from a single tertiary referral center which may limit generalizability to other settings. The patient population and spectrum of organisms in our hospital and region might differ from other U.S. hospitals or other countries. Third, we excluded culture-negative cases of presumed endogenous endophthalmitis from our analysis. By limiting the study to culture-proven infections, we increased the certainty regarding causative organisms and focused our conclusion on definitively diagnosed cases. However, this approach excluded a subset of patients with clinically suspected endogenous endophthalmitis where no organism was isolated. Those culture-negative cases may have yielded different outcomes or may have been caused by fastidious organisms not captured in our series, providing unique data. Finally, the sample size of 50 eyes is relatively small. This limits the statistical power to detect subtle differences in outcomes and precluded any meaningful subgroup analysis.

In summary, *Candida* species remains the most common isolate in cases of endogenous endophthalmitis. The findings of the present study highlight that endogenous endophthalmitis often presents subacutely, without obvious systemic illness, which can lead to misdiagnoses or delayed treatment. Our cohort experienced a lower mortality rate than previous studies, likely reflecting the benefits of evolving systemic therapies and aggressive multidisciplinary care. Endogenous endophthalmitis continues to pose a diagnostic and therapeutic challenge. It is imperative that physicians maintain a high clinical suspicion for this condition in patients with known risk factors who develop atypical intraocular inflammation.

## Data Availability

All data generated or analyzed during this study are included in this published article.
